# Stabilized diverse HIV-1 envelope trimers for vaccine design

**DOI:** 10.1080/22221751.2020.1745093

**Published:** 2020-04-07

**Authors:** Qian Wang, Bingting Ma, Qingtai Liang, Angqi Zhu, Hua Wang, Lili Fu, Xiaoxu Han, Xuanling Shi, Ye Xiang, Hong Shang, Linqi Zhang

**Affiliations:** a Comprehensive AIDS Research Center and Center for Global Health and Infectious Diseases, Beijing Advanced Innovation Center for Structural Biology, Collaborative Innovation Center for Diagnosis and Treatment of Infectious Diseases, Department of Basic Medical Sciences, School of Medicine, Tsinghua University, Beijing, People’s Republic of China; b Department of Cancer Immunology and Virology, Dana-Farber Cancer Institute, Department of Microbiology, Harvard Medical School, Boston, MA, USA; c Center for Global Health and Infectious Diseases, Collaborative Innovation Center for Diagnosis and Treatment of Infectious Diseases, Beijing Advanced Innovation Center for Structural Biology, Department of Basic Medical Sciences, School of Medicine, Tsinghua University, Beijing, People’s Republic of China; d Key Laboratory of AIDS Immunology of the Ministry of Health, Department of Laboratory Medicine, No. 1 Hospital of China Medical University, Shenyang, People’s Republic of China

**Keywords:** HIV-1 Env, subtype CRF01_AE, uncleaved prefusion-optimized (UFO) trimer, SOSIP trimer, vaccine design

## Abstract

One of the major goals in HIV-1 vaccine development is to achieve properly folded and stabilized envelope glycoprotein (Env) trimers that mimic the native Env on the mature virion. Here, we design and characterize uncleaved prefusion-optimized (UFO) trimers for 12 Envs currently circulating in China. Biochemical and biophysical characterization of these UFO trimers identified two subtype B/Bʹ Envs, CNE6 and MG13, which exhibited the highest trimer content and stability at a level comparable to the subtype A reference, BG505. Replacing the gp41 ectodomain (gp41_ECTO_) of CRF01_AE trimers with that of CNE6, MG13, and BG505 resulted in chimeric constructs with significantly improved trimer content and stability. Negative-stain electron microscopy (EM) confirmed the structural integrity of these chimeric UFO trimers with CNE6 gp41_ECTO_. Antibody binding assays showed that the chimeric trimers shared similar antigenic profiles to those with their original gp41_ECTO_ domains. Our results thus revealed the intrinsic differences among HIV-1 Envs of diverse origins and the critical role of gp41_ECTO_ in stabilizing the trimeric spike. By taking advantage of naturally stable Envs, gp41_ECTO_ swapping may represent a universal approach for the generation of stable trimers with the desired structural and antigenic properties for downstream in vivo evaluation and vaccine development.

## Introduction

Since its discovery in 1981, human immunodeficiency virus type I (HIV-1) has posed a significant threat to human health [1,2]. While antiretroviral therapy has achieved sustained viral suppression and reduced viral transmission, there is no vaccine available to prevent HIV-1 infection and no treatment that can completely eliminate the viruses after infection. The Env of HIV-1 is the major target for vaccine development as it mediates viral entry into host cells. As the sole viral protein on the surface of mature virions, HIV-1 Env can be recognized by neutralizing antibodies [3,4]. Synthesized as a gp160 precursor, the Env is processed into the receptor-binding subunit, gp120, and the fusion-mediating transmembrane subunit, gp41. Through noncovalent interactions, gp120 and gp41 form a heterodimer, which further assembles into a trimeric Env complex on the virion surface [5–7].

One of the most promising approaches to HIV-1 vaccine design is focused on the generation of soluble and stable Env trimers that mimic the native viral spikes on mature virions [8,9]. The underlying hypothesis is that, with structural features closely resembling those of prefusion viral spikes, such Env immunogens will elicit an effective antibody response capable of recognizing and neutralizing incoming live HIV-1. Broadly speaking, three designs have been explored to generate such Env trimers, including SOSIP [10–13], native flexibly linked (NFL) [14,15], and UFO [16,17] trimers. The structural information obtained from high-resolution X-ray crystallography and cryo-electron microscopy (cryo-EM) [6,7,15,17,18] has proven crucial to the development and optimization of native-like trimers [8]. All three designs aim to stabilize the Env trimer in a prefusion conformation, thus allowing preferential recognition by bNAbs over non-bNAbs [11,14,16]. While all three trimer designs contain a truncated gp41_ECTO_ at position 664 preceding the transmembrane region to improve solubility, they differ in several regions including the furin cleavage site between gp120 and gp41. The SOSIP gp140 construct contains key mutations that can enhance the cleavage (R6) of the Env precursor and connect the resulting gp120 and gp41 subunits with a covalent disulphide bond between A501C and T650C (SOS), in addition to an I559P mutation (IP) in the HR1 region of gp41_ECTO_ [11]. The NFL and UFO trimers, however, are cleavage-independent and contain a flexible glycine/serine linker (G_4_S) in place of the cleavage site. While the NFL design retains the I559P mutation, the UFO design combines a truncated, redesigned HR1 with the SOS mutation [14,16]. All three strategies appear to produce trimers with antigenic properties and morphologies largely consistent with the native Env complex [11,14,16]. The potential of these trimer platforms for HIV-1 vaccine development is currently under investigation [17,19,20].

In this study, we seek to generate a diverse array of native-like Env trimers based on the UFO design to faciliate the evaluation of immunogenicity, as UFO requires the least number of additional mutatons when applied to non-BG505 Envs [16,17]. We first produced UFO trimers for 12 Envs from the three major HIV-1 subtypes (CRF01_AE, B/Bʹ, and CRF07_BC) currently circulating among infected patients in China. Size exclusion chromatography (SEC) profiles showed that two UFO trimers derived from MG13 and CNE6 exhibited the highest trimer yield and homogeneity, comparable to that of the subtype A reference, BG505. For MG13, CNE6, and BG505, UFO trimers showed greater thermal stability than their respective SOSIP trimers, although they displayed similar antigenic patterns. Next, we replaced the gp41_ECTO_ of less stable subtype CRF01_AE UFO trimers with gp41_ECTO_ from MG13, CNE6, or BG505. The resultant chimeric UFO trimers showed improved yield, purity, and thermal stability, but similar antigenic profiles to their respective UFO trimers containing the wildtype gp41_ECTO_. In contrast, reciprocal substitutions with gp41_ECTO_ of less stable CRF01_AE UFO trimerss reduced the yield and stablity of MG13, CNE6, and BG505 trimers. Furthermore, negative-stain EM demonstrated that chimeric UFO constructs with CNE6 gp41_ECTO_ form trimeric structures. Collectively, our results provide further evidence that gp41_ECTO_ is the main source of Env metastability and indicate that the gp41_ECTO_ of MG13 and CNE6, in addition to BG505 [17], can be utilized to generate chimeric UFO trimers with desired stability and antigenicity. This gp41 swapping approach will rapidly expand the pool of native-like Env trimers for structural analysis and immunogen design in HIV-1 vaccine development.

## Materials and methods

**Antibodies** Antibodies recognizing the CD4 binding site (N6, VRC01, VRC03, VRC07, NIH45-46, 3BNC60, 3BNC117, 12A12, VRC-CH31, VRC-PG04, VRC-PG20, and b6), the V1V2 apex (PG9, PG16, PGT145, VRC26.25, and PGDM1400), the glycan V3 (10-1074, PGT121, PGT128 and PG135), OD-glycan (2G12), fusion peptide (PGT151 and VRC34.01), the interface between gp120 and gp41 (35O22), the membrane proximal external region (2F5, 4E10, and 10E8) of gp41, the CD4 induced epitope (17b and A32), and exposed V3 (39F, 447-52D, and 19b) were used. An Ebola virus-specific antibody 2G4 and influenza virus-HA-specific antibody 3C11 were used as negative controls. Some of these antibodies came from NIH AIDS Reagent Program, or were kindly provided by Drs. John Mascola of the Vaccine Research Center at the NIH, Michel Nussenzweig at Rockefeller University, Barton Haynes at Duke University, Denis Burton at the Scripps Research Institute, and Wayne Koff at the Human Vaccine Project. For some antibodies with published crystal structures and sequences, the variable genes were synthesized and cloned into human IgG1 expression vectors provided by Michel Nussenzweig at Rockefeller University. The full-length IgG1 was expressed by co-transfecting paired heavy- and light-chain plasmids at a ratio of 1:1 using FectoPRO™ DNA Transfection Reagent (WVR) into Expi293 cells (Invitrogen). Antibodies secreted into the culture supernatant were purified 5 days after transfection by affinity chromatography using Protein A columns following the manufacturer’s instructions (Thermo Scientific). Soluble CD4 encoding the first two domains of human CD4 fused to immunoglobulin G Fc (CD4-Ig) was expressed, purified, and quantified as for the antibodies above.

**Cell lines** HEK293 T and TZM-bl cells were propagated in Dulbecco’s modified Eagle medium (DMEM) supplemented with 10% foetal bovine serum (FBS), penicillin, streptomycin, and L-glutamine. Expi293 cells and 293F cells were incubated with Expi293^TM^ Expression Medium and FreeStyle 293 Expression Medium respectively, and supplemented with penicillin and streptomycin in a shaker incubator at 37°C, with 125 rpm and 8% CO_2_.

**Construct design** The UFO and SOSIP constructs have been previously described [11,16]. In brief, the following changes were introduced into an HIV-1 Env to create a UFO construct: mutations A501C and T605C to form a disulphide bond; the cleavage site sequence REKR between gp120 and gp41 changed to a (G_4_S)_2_ linker; the HR1 bend (residues 547-569) replaced with a redesigned segment (NPDWLPDM); and truncation at residue 664 and addition of a C5 tag (GSAPTKAKRRVVQREKR) at the C-terminus. The following changes were incorporated into an HIV-1 Env to create a SOSIP construct: mutations A501C and T605C; REKR to RRRRRR at the cleavage site between gp120 and gp41; I559P in the HR1 bend of gp41_ECTO_; truncation at residue 664 and addition of a C5 tag sequence at the C-terminus.

**Expression and purification of Env trimers** Env trimers were produced by transient transfection of engineered expression vectors into 293F cells with poly-ethyleneimine (PEI) at a density of 2.5–3.0 × 10^6^/ml [11,16]. For UFO trimers, 600 µg of Env plasmid in 25 ml of Opti-MEM transfection medium (Life Technologies, CA) was mixed with 2 ml of PEI (1.0 mg/ml) in 25 ml of Opti-MEM. For SOSIP trimers, 600 µg of Env plasmid along with 150 µg furin plasmid in 25 ml of Opti-MEM transfection medium (Life Technologies, CA) was mixed with 2.5 ml of PEI (1.0 mg/ml) in 25 ml of Opti-MEM. After incubation for 20 min, the DNA-PEI complex was added to 600 ml 293F cells. Culture supernatants were collected 4 days after transfection, centrifuged at 8000 rpm for 30 min, and filtered using 0.45-mm filters. The Env proteins were extracted from the supernatants using a *Galanthus nivalis* lectin (GNL) column (Vector Labs). The bound proteins were eluted with 5 ml PBS containing 500 mM NaCl and 1 M methyl-α-D-mannopyranoside and then purified by SEC on a Superdex 200 Increase 10/300 GL column. We then decomposed the SEC profiles into aggregates, trimer, and dimer/monomer fractions and calculated percentage of each fraction using the software UNICORN. Protein concentrations were determined using UV_280_ absorbance [21].

**Neutralization assay** Pseudoviruses were generated by co-transfecting the Env expression vectors with the luciferase gene containing backbone pNL4-3R-E with PEI into HEK293 T cells as described previously [22]. Pseudovirus supernatants were collected 48 h post-transfection and the viral titres were measured as luciferase activity (RLU) in TZM-bl cells. For each pseudovirus, the TCID_50_ was calculated and then the virus was aliquoted and stored at −80°C until use. Neutralizing assays were performed by adding 200 TCID_50_ of pseudovirus into serial dilutions of purified antibodies or CD4-Ig. The mixture was dispensed onto a 96-well plate in triplicate and incubated for 1 h at 37°C. Approximately 2 × 10^4^ TZM-bl cells with 3 μg DEAE-Dextran per well were then added. The cultures were maintained for an additional 72 h at 37°C before luciferase activity was measured. Neutralizing activity was calculated from the reduction in luciferase activity compared with controls. IC_50_ values were determined by fitting five-parameter dose–response curves in GraphPad Prism (GraphPad Software Inc.).

**D7324-capture ELISA** Antigenicity of UFO and SOSIP.664 trimers was determined by sandwich ELISA as described [11]. 96-well plates were coated with C5 tag-specific antibody D7324 (Aalto Bioreagents, Dublin, Ireland) at 10 μg/ml (100 μl/well). After blocking with 300 μl of 5% (w/v) nonfat milk in PBS followed by a washing step, D7324-tagged trimer proteins were added at 500 ng/ml (100 μl/well) for 2 h. Unbound Env proteins were removed by washing three times with PBST (PBS with 0.5‰ Tween 20). PBS plus 2% skimmed milk (200 μl/well) was added to block non-specific protein-binding sites. Antibodies in PBS/2% skimmed milk (100 μl/well) were then added and incubated for 2 h followed by 3 washes with PBST. HRP conjugated anti-human IgG antibody (Promega) diluted at 1:5000 (100 μl/well) was added and incubated for 45 min. After washing three times with PBST, TMB substrate (100 μl/well) was added and the reaction was stopped by addition of 1M H_2_SO_4_ (50 μl/well). Absorbance was measured at 450 nm.

**Measurement of thermal denaturation temperature (T_m_ value)** GNL-purified UFO and SOSIP trimer proteins were exchanged into PBS using centrifugal filter units (Millipore) and were diluted to 0.5–1 mg/ml. Proteins were injected into a MicroCal VP-Capillary DSC instrument (Malvern) and melting temperature was probed at a scan rate of 90°C/h from 20°C to 90°C. Data were analysed using the VP-Capillary DSC automated data analysis software. T_m_ values are for the tallest peak of trimer proteins.

**SDS-PAGE and BN-PAGE** UFO proteins were analysed by both 10% SDS-PAGE (Cwbio, CW0022) and 8% BN-PAGE [23]. For SDS-PAGE, UFO proteins were first denatured with 5% [v/v] β-mercaptoethanol at 95°C for 10 min and then run at 120 V until the dye front reached the bottom. When performing BN-PAGE, samples were treated with a solution containing 10% glycerin and 0.5% Coomassie blue G250, and then run for 3 h at 150 V.

**Negative-stain EM** Parental and chimeric UFO trimers were analysed by negative-stain EM. 3 μl of the purified Env proteins (0.05 mg/ml) were applied to a pre-glow discharged carbon-coated EM grid (Beijing XinxingBraim Technology Co., Ltd.) and then stained with 2% uranyl acetate for 1 min. Micrographs were collected on a 120-kVFEI Tecnai Spirit microscope at a nominal magnification of 49,000 (pixel size is 2.3 Å). About 100,000 particles from the 40 raw micrographs were boxed using the EMAN2 program e2boxer.py [24]. The extracted particles selected by the box coordinates from e2boxer.py were applied for the reference-free 2D class averages by using RELION1.4 [25]. Then, the 90 × 90 pixel boxes were utilized for 2D classification. Aberrant particles were excluded and only the ones with reliable quality were further used for analysis.

## Results

**Figure 1.**
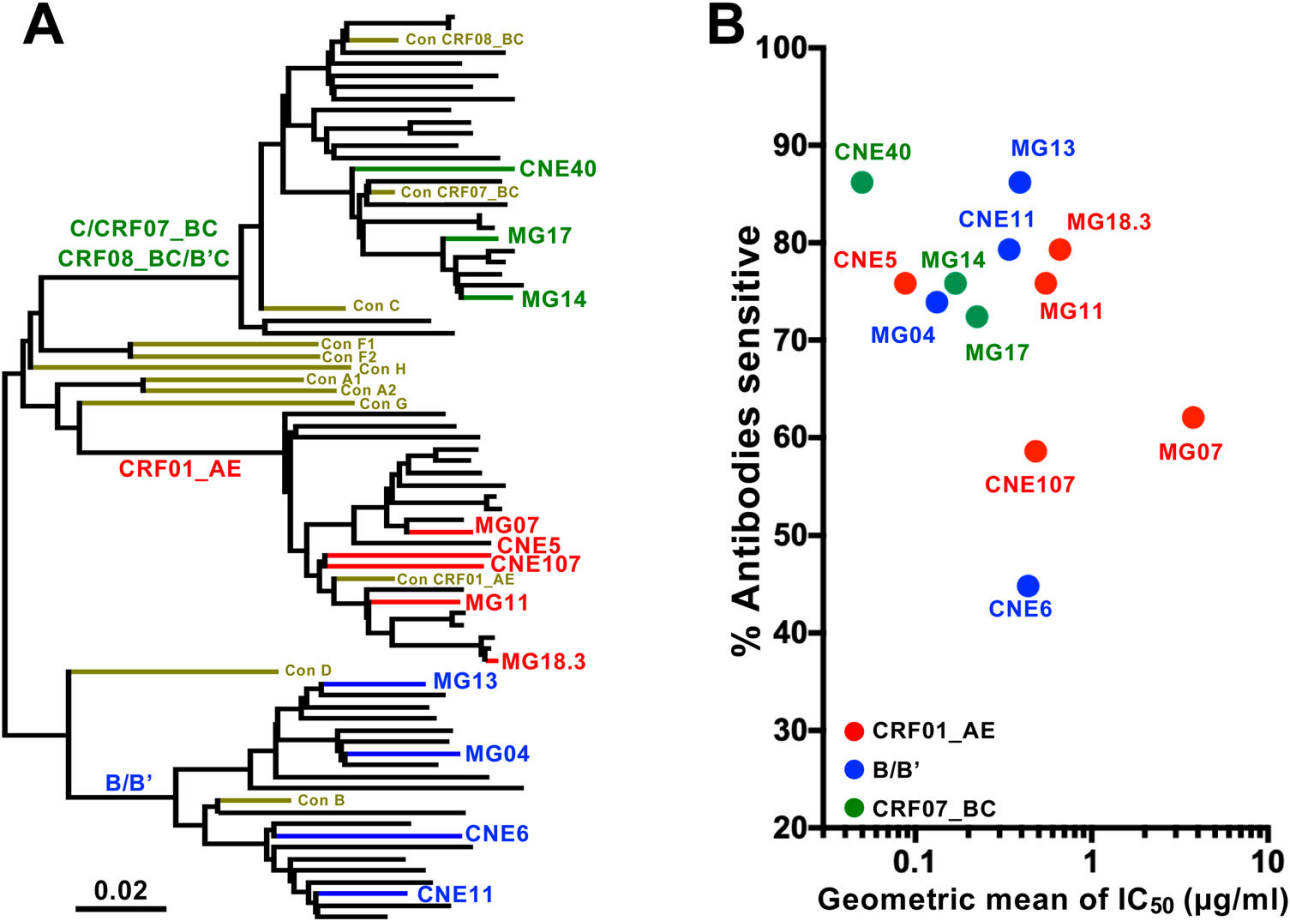
Selection of envelope clones for UFO construction. A. Neighbor-joining tree depicting the genetic relationships among the selected acute (MG) and chronic (CNE) envelope clones circulating in China. Each clone’s branch is drawn in proportion to their relative genetic distance. Red branches are those derived from subtype CRF01_AE, blue are from subtype B/Bʹ, green are from subtype CRF07_BC, and brown are consensus sequences obtained from the HIV Database (www.hiv.lanl.gov). B. Neutralization sensitivity of pseudoviruses carrying the selected Envs to a panel of 27 HIV-1 bNAbs and CD4-Ig. The Y axis shows the percentage of antibodies able to neutralize one pseudovirus (% Antibodies sensitive) and X axis shows the corresponding geometric mean of IC50s. Average data from two independent experiments is presented. Dots are coloured as in A.

**Selection of HIV-1 envelope clones for the construction of Env UFO trimers.** We previously characterized a large panel of full-length HIV-1 envelope clones from infected individuals in China. They were collected during both acute and chronic phases of infection and are widely distributed among the three major genetic clusters: subtype CRF01_AE, subtype B/Bʹ, and subtype CRF07/CRF08_BC [26]. When pseudoviruses bearing these Envs were exposed to subtype-specific plasma pools and bNAbs, we observed substantial differences in neutralization sensitivity [26]. Some have been selected for the Global Panel of HIV-1 Env Reference Clones under the NIH-AIDS Reagent Program (Cat #12670) based on their unique genotypic and phenotypic features [27]. Here, we selected a total of 12 envelope clones from the panel for the construction of UFO trimers. Criteria such as subtype representation, ease of pseudovirus production, and general sensitivity to neutralization by bNAbs over non-bNAbs were considered in the selection (Figure 1 and Table S1). Of the 12 clones, five (MG18.3, MG11, CNE5, CNE107, and MG07) were subtype CRF01_AE, four (CNE11, CNE6, MG3, and MG04) were subtype B/Bʹ, and three (MG14, MG17, and CNE40) were subtype CRF07_BC (Figure 1(A)). Their neutralization profiles with respect to CD4-Ig, 27 bNAbs, and 6 non-bNAbs were characterized either by IC_50_ geometric means or by percentage of bNAbs which were able to neutralize one virus (Figure 1(B) and Table S1). Of the 12 clones, nine were sensitive to neutralization by over 70% of the bNAbs. Among the remaining three, CNE107 and MG07 were sensitive to about 60% while CNE6 was sensitive to less than 50% of bNAbs tested. In fact, CNE6 is a broadly resistant clone to CD4-binding site bNAbs that we recently identified [28]. As neutralizing sensitivity may not be correlated with trimer stability, all 12 Env clones were advanced to the UFO cosntruct design to facilitate further comparison and validation.

**Figure 2.**
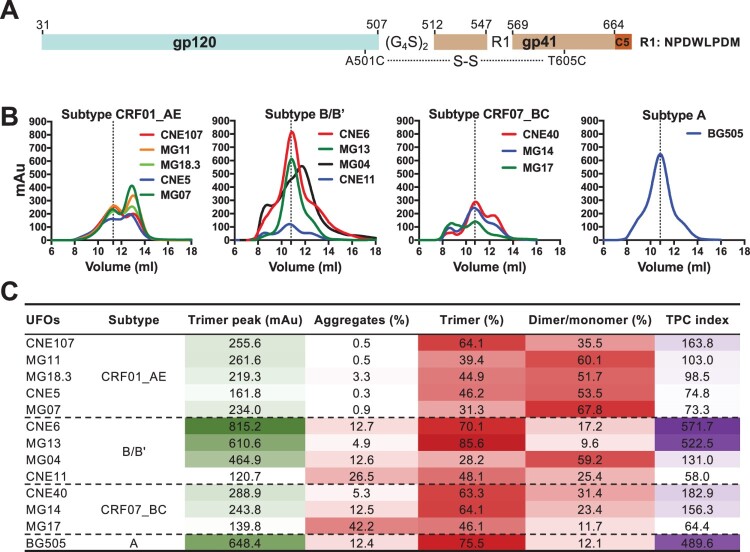
Construction and characterization of UFO trimers. A. Construction scheme of UFO trimers. B. SEC profiles of UFO trimers derived from subtype CRF01_AE, B/Bʹ, CRF07_BC, together with BG505 from subtype A. The dotted line highlights the peak of the trimer. Representative data from one of two independent experiments is shown. C. Ultraviolet absorbance at 280 nm (UV280) of the UFO trimer peak, and corresponding percentage of aggregates, trimer and dimer/monomer decomposed from the SEC profile in B. Trimer peak and content index (TPC index) is calculated by multiplying the actual values of trimer peak (mAu) and trimer percentage. The darker colour represents the higher value.

**Construction and characterization of UFO trimers.** Twelve selected envelope clones along with BG505 were genetically engineered into the UFO format as previously reported [16]. The cleavage site between gp120 and gp41 was first replaced by a (G_4_S)_2_ linker. The N terminus of the HR1 region was then shortened and replaced with a computationally optimized 8-residue fragment to reduce envelope metastability. Finally, an extra disulphide bond was introduced between A501C and T650C to hold gp120 and gp41 together and to stabilize the trimeric protein in a pre-fusion conformation (Figure 2(A) and Figure S2). All constructs were confirmed through sequencing before being transfected into 293F cells for trimer expression. Secreted UFO trimers in the cell culture supernatant were purified in two consecutive steps using a GNL column and a Superdex 200 10/300 GL column, respectively. The size exclusion chromatography (SEC) profiles for each UFO trimer are shown according to their subtype assignment (Figure 2(B)). Significant differences were found in trimer peak (mAu) and trimer content (%) among different clones within and between different subtypes. Two clones (CNE6 and MG13) from subtype B/Bʹ demonstrated the highest trimer peak and content among the 12 selected clones, at a similar level to the reference standard, BG505 (Figure 2(B)). The UV_280_ value of the trimer peak reached 815.2 mAu for CNE6 and 610.6 mAu for MG13. The trimer content approached as high as 70.1% for CNE6 and 85.6% for MG13 (Figure 2(C)). Consistent with earlier reports, BG505 UFO trimer was highly produced with a trimer peak UV_280_ value of 648.4 mAu and a rate of proper trimer folding of about 75.5%. In particular, when we analysed the trimer peak and content together to create a trimer peak and content index (TPC index), the superiority of CNE6, MG13, and BG505 became more evident, showing TPC values of 571.7, 522.5, and 489.6, respectively (Figure 2(C)). The molecular weight (MW) of the trimer fraction was measured for CNE6, MG13 and BG505 UFO trimers by electrophoresis. SDS-PAGE revealed uncleaved monomers of approximately 140KD (Figure S1A), while BN-PAGE showed characteristic trimer bands (Figure S1B), confirming that they were properly folded, native-like gp140 trimers consistent with previous reports [16,17]. The structural integrity of CNE6, MG13, and BG505 UFO trimers was further verified by negative-stain EM, with the native-like trimer morphology observed for the majority of the Env proteins after purification (Figure S1C). In contrast, the TPC index was well below 200.0 for the remaining Envs, which also contained aggregates, dimers, and monomers (Figure 2(C)). Of note, the trimer content for subtype CRF01_AE was disproportionally affected by the formation of dimers and monomers than by aggregation (Figure 2(C)), suggesting a unique feature associated with Envs of the CRF01_AE origin in their gp140 folding and trimerization.

**Figure 3.**
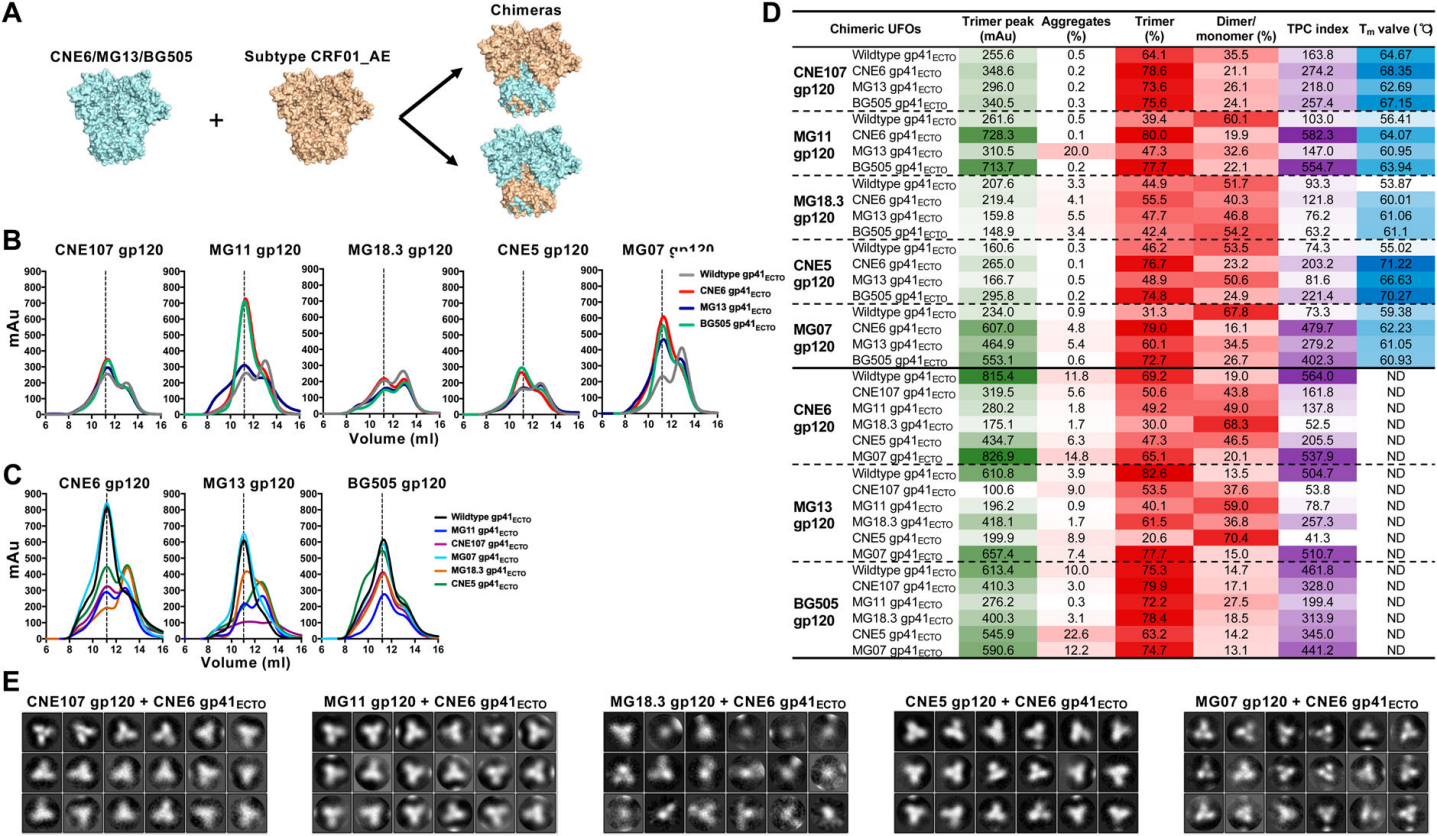
Construction and characterization of chimeric UFO trimers A. Construction scheme of chimeric UFO trimers. B. C. SEC profiles of the chimeric UFO trimers compared with their parental ones. The chimeric UFO trimers that carry the gp41ECTO or gp120 from CNE6, MG13, or BG505 are shown in B and C, respectively. Representative data from one of two independent experiments is shown. D. UV280 value of the chimeric and wildtype UFO trimer peak, and corresponding percentage of aggregates, trimer and dimer/monomer decomposed from the SEC profile in B and C. TPC index was calculated by multiplying the actual values of trimer peak (mAu) and trimer percentage. The darker colour represents the higher value. Tm value, thermal denaturation midpoint value; WT, wildtype; ND, not done. E. 2D classifications from the negative-stain EM of five chimeric UFO trimers with CNE6 gp41ECTO. Significant portions of CNE5, CNE107 and MG11 chimaeras appear to be native-like trimers. MG07 chimaera and MG18.3 chimaera, however, demonstrated substantial irregular morphologies.

**Gp41 is the main source of Env metastability**. In a previous study, gp41 was identified as the main source of HIV-1 metastability by replacing gp41_ECTO_ of diverse Env trimers with BG505 gp41_ECTO_ containing the UFO design [17]. Here, we examined this hypothesis with a large panel of CRF01_AE Envs. Since significant variation in the TPC index was found among UFO trimers, we sought to identify the potential genetic determinant(s) for such variability. To this end, we selected those UFO trimers with the highest TPC index (CNE6, MG13, and BG505) and genetically swapped their gp41_ECTO_ or gp120 with those with low TPC indexes from CRF01_AE (Figure 3(A)). The resultant chimeric clones were confirmed through sequencing and transfected into 293F cells for transient expression. Produced Env proteins were purified from the cell culture supernatant by the two step process described above, and generated the SEC profiles shown in Figure 3(B,C). The chimeric clones bearing a CNE6, MG13, or BG505 gp41_ECTO_ showed the most significant improvement in trimer peak and content, although the degree of such improvement varied from clone to clone (Figure 3(B)). The TPC index increased from 73.3–163.8 for the wildtype clones to 121.8–582.3 for the chimeric clones with CNE6 gp41_ECTO_ (Figure 3(D)). The most notable effect was found for MG11 and MG07, for which the TPC index increased by 5.4- to 6.5-fold (Figure 3(D)). However, the gp41_ECTO_ of MG13 appeared to be less effective than that of CNE6 and BG505 (Figure 3(D)). In addition, the thermal stability of the chimeric UFO trimers was determined by thermal denaturation midpoint. Nearly all of the chimeric trimers yielded higher T_m_ values than their corresponding wildtype constructs, with a more than 11°C increase in T_m_ observed for CNE5 upon gp41_ECTO_ swapping (Figure 3(D)). Conversely, the chimeric clones carrying the gp120 region of CNE6, MG13 and BG505 failed to improve the TPC index for CRF01_AE Envs (Figures 3(C,D)). These chimeric clones, with the exception of those derived from MG07, showed a reduced TPC index compared to the wildtype CNE6, MG13 and BG505 (Figure 3(D)). Taken together, our results indicate that gp41_ECTO_ plays a more important role than gp120 in Env metastability, consistent with the previous study where such effect was examined using primarily BG505 gp41_ECTO_ [17]_._

Since the chimeric UFO trimers with a CNE6 gp41_ECTO_ showed higher trimer content and thermal stability, they may be useful for trimer-based vaccine development. To faciliate this effort, we characterized the structures of these chimeric UFO trimers by negative-stain EM. About 100,000 particles were selected by the EMAN2 program e2boxer.py and then used for 2D class averages. Indeed, most of the CNE5, CNE107, and MG11 chimeric UFO trimers appeared to be native-like, characterized by the three-bladed propeller morphology (Figure 3(E)). However, the MG07 and MG18.3 chimeric UFO trimers displayed various non-trimeric forms. Furthermore, there appeared to be a correlation between trimer morphology and thermal stability, as CNE5, CNE107, and MG11 chimaeras yielded higher T_m_ values than MG07 and MG18.3 chimaeras (Figure 3(D)).

**Figure 4.**
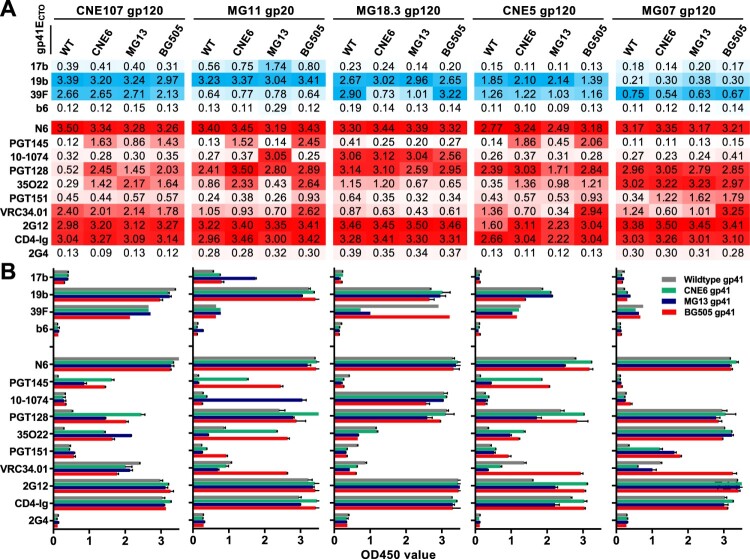
Antigenic profiles of the chimeric UFO trimers. The chimeric UFO trimers carrying the gp120 of selected CRF01_AE Envs and the gp41ECTO of CNE6, MG13, and BG505 are shown. ELISA readouts against the panel of antibodies are summarized either in actual OD450 values (A) or in histogram (B). Non-bNAbs are coloured in blue while bNAbs are in red in (A). The darker colour represents the higher value. Representative data from one of two independent experiments is shown. WT, wildtype.

**Similar antigenicity between the chimeric and wildtype UFO trimers.** Next, the effect of gp41_ECTO_ swapping on trimer antigenicity was investigated by ELISA. To this end, we compared the binding activity of chimeric CRF01_AE UFO trimers featuring gp41_ECTO_ from CNE6, MG13, or BG505 with their respective wildtype UFO trimers against a panel of 13 antibodies and CD4-Ig. These antibodies included four non-neutralizing antibodies (non-NAbs) targeting the CD4 induced epitope (17b), exposed V3 loop (19b and 39F), the CD4 binding site (b6), as well as eight bNAbs targeting the CD4 binding site (N6), V1V2 (PGT145), V3 glycan (10-1074 and PGT128), gp120-gp41 interface (35O22), fusion peptide (PGT151 and VRC34.01), and OD-glycan (2G12). CD4-Ig, an FC-fusion protein containing the first two domains of human CD4 linked to immunoglobulin G Fc, and antibody 2G4, which targets the surface glycoprotein of the Ebola virus, were used as positive and negative controls, respectively. Overall, similar antigenic profiles were observed between all chimeric UFO trimers and their corresponding wildtype UFO trimers, although some minor differences were identified (Figure 4). For example, chimeric UFO trimers with higher TPC indexes, MG11 and MG07 (Figure 3(D)), demonstrated either improved or similar binding profiles to bNAbs compared to their respective wildtype UFO trimers (Figure 4(A)). In particular, MG11 chimeric UFO trimers with the BG505 or CNE6 gp41_ECTO_ exhibited improved binding to bNAbs PGT145 and 35O22, indicating that a more stable gp41_ECTO_ may help preserve those conformational epitopes at the V2 apex and the gp120-gp41 interface, respectively (Figure 4). Interestingly, the MG11 chimeric UFO trimer with the MG13 gp41_ECTO_ instead showed improved binding to the V3 glycan antibody 10–1074 and the non-NAb 17b that recognizes a CD4-induced epitope, suggesting that replacing wildtype gp41_ECTO_ may impact both bNAb and non-NAb epitopes. Conversely, the MG07 chimeric UFO trimers showed minimal antigenic differences from the wildtype UFO trimer. Notably, MG11, CNE5, and MG07 chimeric UFO trimers with the BG505 gp41_ECTO_ displayed enhanced binding to the fusion-peptide specific antibody VRC34.01, consistent with neutralization data where the pseudovirus bearing BG505 Env was sensitive to VRC34.01 while those bearing MG11, CNE5, and MG07 Env were resistant (Table S1 and HIV database [29]).

Despite relatively lower TPC indexes (Figure 3(D)), the chimeric UFO trimers from CNE107, MG18.3, and CNE5 demonstrated similar antigenic trends when compared with their respective wildtype UFO trimers (Figure 4(A)). Similarly to the MG11 chimeric UFO trimers, CNE107 and CNE5 chimeric trimers with the BG505 or CNE6 gp41_ECTO_ showed improved binding to PGT145 and 35O22 (Figure 4). Nonetheless, this suggests that conformational epitopes at the V2 apex and gp120-gp41 interface are maintained by more stable gp41_ECTO_ regions (Figure 4). Finally, the chimeric UFO trimers from MG18.3 displayed binding profiles similar to the wildtype, except for those with CNE6 or MG13 gp41_ECTO_, for which a reduction in binding to the V3 non-NAb 39F was observed (Figure 4).

Altogether, these results demonstrate that utilization of gp41_ECTO_ derived from inherently stable Envs such as CNE6, MG13, and BG505 for gp41_ECTO_ swapping will not only improve trimer formation but also preserve the overall antigenic properties of heterologous Envs. This strategy represents a promising approach to transforming less stable Env trimers into stable ones with structural and antigenic properties that are suitable for trimer-based vaccine development.

**Figure 5.**
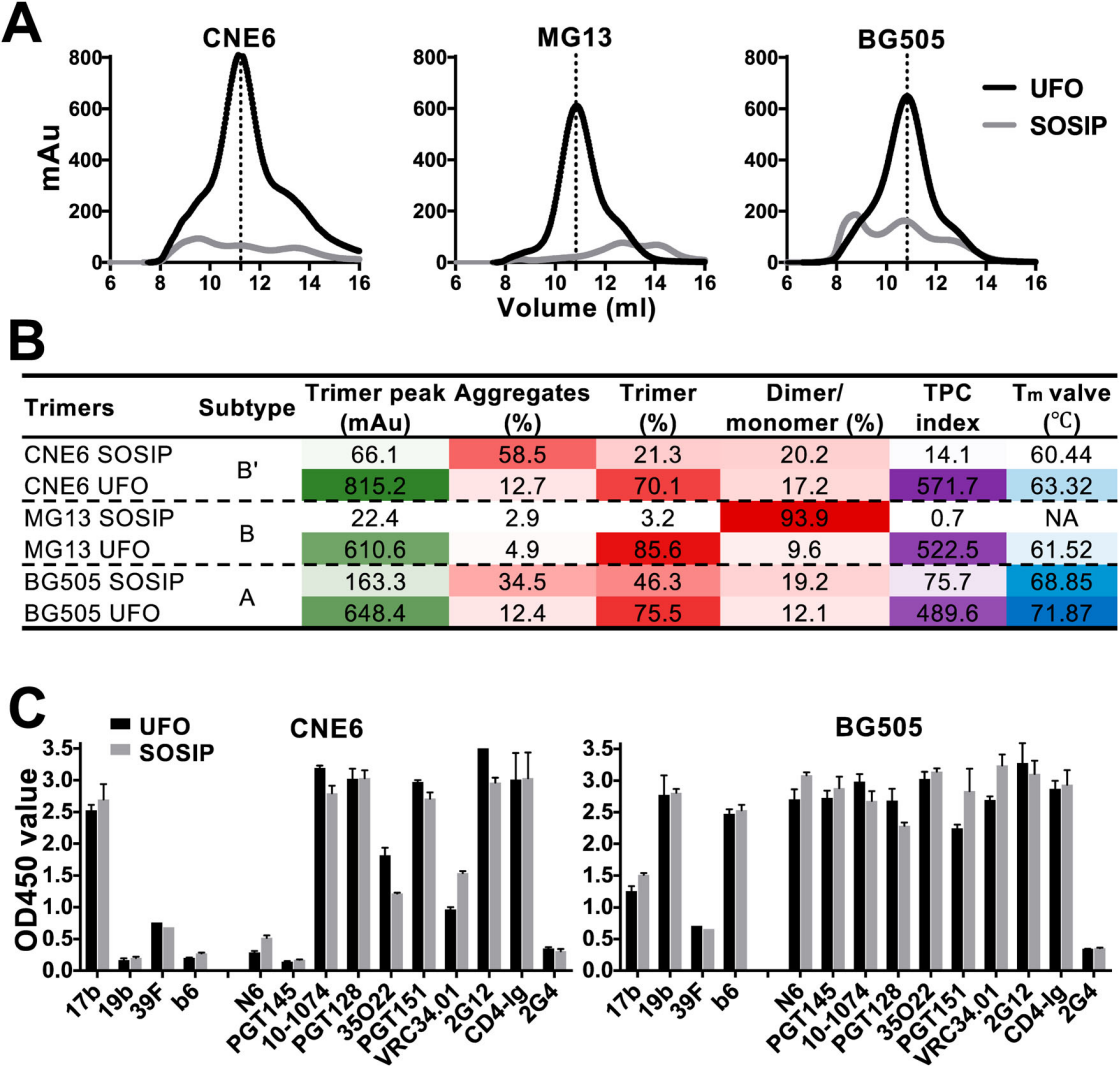
Comparison in biophysical and antigenic properties between UFO and SOSIP trimers. A. SEC profiles of 293F-expressed and GNL-purified UFO and SOSIP trimers. Representative data from one of two independent experiments is shown. B. UV280 value of the trimer peak, and corresponding percentage of aggregates, trimer and dimer/monomer decomposed from the SEC profile in A. TPC index is calculated by multiplying the actual values of trimer peak (mAu) and trimer percentage. The darker colour represents the higher value. Tm value, thermal denaturation midpoint value. NA, not available. C. Comparison in antigenicity between the UFO and SOSIP trimers. ELISA readouts against the panel of antibodies are summarized in histogram. Representative data from one of two independent experiments is shown.

**A comparative analysis of UFO and SOSIP in trimer formation.** Following structural and antigenic characterization, the effect of the specific trimer design was assessed for UFO and SOSIP. This comparative analysis was conducted to determine if biochemical and biophysical features would be maintained when the UFO trimers were converted to the SOSIP format. The UFO trimers of three Envs (CNE6, MG13, and BG505) that demonstrated the highest TPC indexes among the tested Envs were selected to facilitate this comparison. We then produced UFO and SOSIP trimers and obtained their structural and antigenic profiles under identical experimental conditions. The SEC profiles showed that UFO outperformed SOSIP in terms of trimer formation (Figure 5(A)). The trimer peak of UFO trimers reached 815.2 mAu for CNE6, 610.59 mAu for MG13, and 648.43 mAu for BG505, whereas that of the corresponding SOSIP trimers only reached 66.1, 22.4, and 163.3 mAu, respectively (Figure 5(B)). In addition, higher trimer content was observed for UFO trimers. In the case of CNE6, the UFO construct exhibited about 70.1% trimer content as compared to 21.3% for the SOSIP construct. A similar trend was also observed for MG13 and BG505 trimers (Figure 5(B)). Trimer formation was further assessed for the two trimer designs using the TPC indexes. The TPC indexes increased from 0.7-75.5 for SOSIP trimers to 489.5-571.7 for UFO trimers, which translated to 39.7-, 746.4-, and 6.5-fold changes for CNE6, MG13, and BG505, respectively (Figure 5(B)). Furthermore, UFO trimers demonstrated greater thermal stability compared to SOSIP trimers. The T_m_ value showed an increase of 2.88°C for CNE6 and 3.02°C for BG505, while T_m_ could not be determined for MG13 SOSIP trimer due to the extremely low yield. Finally, we compared the antigenic properties of UFO and SOSIP trimers, using the large antibody panel described above, which recognizes the major Env epitopes. Both trimer designs showed similar binding profiles for CNE6 and BG505, although significant differences in antigenicity were observed between Envs (Figure 5(C)). Collectively, the results for these three Envs demonstrated that Envs containing the UFO design may form native-like trimers more effectively than the SOSIP design, although a high degree of similarity in antigenicity was maintained between the two designs.

## Discussion

After infection in vivo, HIV-1 rapidly generates viral variants enabling viral escape from immune recognition [30–32]. Common mechanisms of HIV-1 immune evasion include sequence diversity, conformational metastability and the glycan shield [33–35], all of which pose major challenges to HIV-1 vaccine development. Traditional vaccine approaches have thus far failed to overcome the challenge of HIV-1 diversity despite remarkable success against a large array of pathogens with limited variability [36]. It has become increasingly clear that a more rational approach is required to develop protective vaccines against HIV-1. Recent determination of atomic-level structures of HIV-1 Env trimers has defined several major sites and conformational states vulnerable to recognition and neutralization by bNAbs [3]. In particular, Env trimers stabilized in the prefusion, closed conformation have become one of the most promising candidates for downstream vaccine development. These trimers display the broadest accessibility to conserved sites of vulnerability and are thus expected to elicit an antibody response with broad neutralization activity. In fact, several of these Env trimers are being evaluated as vaccine candidates in humans for their safety and immunogenicity [37–40].

In this study, we generated and characterized a diverse repertoire of HIV-1 Env trimers derived from the three major subtypes (CRF01_AE, B/Bʹ, and CRF07_BC) currently circulating among infected patients in China. Of the three leading Env trimer designs (SOSIP, NFL and UFO), we chose UFO largely due to its ability to stabilize diverse Envs in the prefusion conformation with relative ease in construct design and production [16,17]. Among the 12 UFO trimers studied here, each displayed distinct features in trimer formation and stability. The UFO trimers derived from CNE6 and MG13 of subtype B/Bʹ were found to have the highest trimer content and yield, comparable to that of BG505 from subtype A (Figure 2). However, the remaining UFO trimers displayed rather poor trimer formation. Consistent with earlier reports [16,17,41], replacing the gp41_ECTO_ of poorly behaved trimers with that from inherently stable trimers (CNE6, MG13, and BG505) significantly improved trimer content, yield, and stability, while Env antigenicity was largely independent of the gp41_ECTO_ swapping (Figures 3 and 4). In contrast, the impact of substituting the gp120 region was rather small. Finally, the UFO trimers derived from CNE6, MG13, and BG505 exhibited superior trimer content, yield, and stability than the corresponding SOSIP trimers, although the two trimer designs share a high degree of similarity in antigenicity (Figure 5). Taken together, our results demonstrate the intrinsic differences among different Envs and the critical role of gp41_ECTO_ in facilitating trimer formation. Therefore, gp41_ECTO_ swapping utilizing gp41_ECTO_ derived from naturally stable Envs may represent a universal approach for the generation of chimeric trimers with desirable structural and antigenic properties for trimer-based vaccine development. Several findings in our study may prove useful for future HIV-1 vaccine development that are discussed below.

First, HIV-1 Envs, regardless of the design platform used (UFO or SOSIP alike), appear to have differing propensities for trimer formation [12,16]. Subtype CRF01_AE Envs exhibited low trimer content and stability compared to those of other subtypes (Figure 1), suggesting that such differences may be caused by certain genetic features characteristic to the CRF01_AE subtype. Recombination studies among various clones demonstrated that the genetic determinant for trimer formation is more likely encoded by gp41_ECTO_ rather than gp120 [16,17,41]. As CRF01_AE viruses represent one of the major subtypes circulating in southeast Asian countries and are also responsible for 55.8% of newly infected cases among men who have sex with men (MSM) in China [42,43], construction and characterization of Envs from this subtype are critical for downstream vaccine development. Replacing the gp41_ECTO_ of CRF01_AE Envs with gp41_ECTO_ from intrinsically stable Envs such as CNE6, MG13 or BG505 would provide a promising vaccine strategy for this HIV-1 subtype. However, mechanistically, it is unclear whether intrinsic differences between Envs in trimer formation truly reflect the properties of the native trimers, such as the immunogenicity. Recent analysis has demonstrated some degree of difference between recombinant SOSIP trimers and those on the native virion. SOSIP trimers frequently transition into a CD4-bound high-FRET state while Envs on matured virions prefer a more closed conformation and predominantly occupy the unliganded ground-state configuration [44,45]. Additionally, the SOSIP trimers did not bind at all to non-NAbs particularly in gp120 V3 and some CD4bs epitopes, as well as possessed slightly less complex and less extensively processed glycans compared to the same full-length trimer [46–48]. Although no UFO trimer has been characterized using this FRET-based dynamic approach, the Env bearing the UFO design has been proven to display similar antigenicity to its native-like, membrane-bound form [49]. Given the outstanding performance of the UFO design in trimer formation, it will be important to investigate the dynamics of UFO trimers, as well as SOSIP and NFL trimers, in comparison with Envs on matured virions. Such studies will facilitate the selection and optimization of the best strategy for generating trimeric Envs that most closely resemble the native spikes on HIV-1 virions for downstream vaccine development. Additionally, our follow-up studies will aim to dissect the individual residue(s) in the gp41_ECTO_ by mutagenesis in an effort to provide insight into the stabilization of Env trimers, thereby leading to more optimal trimer-based vaccine candidates.

Second, it is well known that for a vaccine candidate optimal antigenicity in vitro does not necessarily translate to superb immunogenicity in vivo [50]. Even if it were possible to create Env trimers with identical structural and antigenic profiles to those on mature HIV-1 virions, it would still be necessary to address the question of which ones, or which combinations thereof, are more likely to trigger bNAb-like responses during vaccination. As both a prolonged antibody maturation process and presence of rare antibody characteristics are required for bNAb development during natural infection in humans, the selection of appropriate Env trimers for immunization with a specifically designed regimen to achieve optimal vaccine response remains challenging. In recent HIV-1 vaccine studies, several immunization strategies have been reported to induce bNAb-like responses in vivo. These strategies include “germline targeting” – design of trimeric Env immunogens to trigger specific germline ancestors of bNAbs [3,51]. In bNAb or bNAb precursor knock-in mice, some of these Env immunogens have shown encouraging results in triggering B-cell receptors and generating neutralizing antibodies against the autologous virus but have failed to elicit a broadly neutralizing response [52–54]. Future optimization may focus on improving Env immunogenicity either through construct modification and/or utilizing novel delivery systems such as live vectors.

In summary, we have identified and characterized a small set of Envs with inherently stable gp41_ECTO_, including CNE6, MG13 and BG505. The gp41_ECTO_ domain derived from these Envs can be used to stabilize diverse Envs of the CRF01_AE subtype, thus presenting a key step towards vaccine development for populations predominantly afflicted by this HIV-1 subtype.

## Supplementary Material

Supplemental Material
