# Supplementary material for: Stabilized diverse HIV-1 envelope trimers for vaccine design
Source: Emerg Microbes Infect. 2020 Apr 7;9(1):775–86. doi: 10.1080/22221751.2020.1745093 (PMC7178897; doi:10.1080/22221751.2020.1745093)
Supplement: Supplemental Material [file TEMI_A_1745093_SM8615.docx]

**Table S1.** Neutralization sensitivity of HIV-1 carrying the selected envelope clones for UFO trimer construction. Representative data of one in two independent experiments is shown. ND, not done.

| **Antibody** | | | **CRF01_AE** | | | | |  | **B/B'** | | | |  | **CRF07_BC** | | | |
| --- | --- | --- | --- | --- | --- | --- | --- | --- | --- | --- | --- | --- | --- | --- | --- | --- | --- |
|  |  |  | **CNE107** | **MG11** | **MG18.3** | **CNE5** | **MG07** |  | **CNE6** | **MG13** | **MG04** | **CNE11** |  | **CNE40** | **MG14** | **MG17** |  |
| **Non bNAbs** | | | | | | | | | | | | | | | | | |
| CD4i | | 17b | >50 | >50 | >50 | >50 | >50 |  | >50 | >50 | >50 | >50 |  | 0.013 | >50 | >50 |  |
|  |  | A32 | >50 | >50 | >50 | >50 | >50 |  | >50 | >50 | >50 | >50 |  | >50 | >50 | >50 |  |
| CD4bs | | b6 | >50 | >50 | >50 | >50 | >50 |  | >50 | >50 | >50 | >50 |  | 0.068 | >50 | >50 |  |
| Exposed V3 | | 39F | >50 | >50 | >50 | >50 | >50 |  | >50 | >50 | >50 | >50 |  | 0.455 | >50 | >50 |  |
|  |  | 447-52D | >50 | >50 | >50 | >50 | >50 |  | >50 | >50 | >50 | >50 |  | 10.042 | >50 | >50 |  |
|  |  | 19b | >50 | >50 | >50 | >50 | >50 |  | >50 | >50 | >50 | >50 |  | 0.146 | >50 | >50 |  |
| **bNAbs** | | | | | | | | | | | | | | | | | |
| CD4bs | | N6 | 0.076 | 0.417 | 0.120 | 0.074 | 1.002 |  | >50 | 0.084 | ND | 0.078 |  | 0.028 | 0.012 | 0.126 |  |
|  |  | VRC01 | 0.788 | 4.544 | 0.494 | 0.194 | 16.530 |  | >50 | 0.365 | 0.191 | 0.166 |  | 0.140 | 0.174 | 0.484 |  |
|  |  | VRC03 | >50 | 2.469 | >50 | 1.886 | 35.920 |  | >50 | 15.305 | 0.025 | >50 |  | 0.042 | 0.067 | 0.144 |  |
|  |  | VRC07 | 0.028 | 0.532 | 0.118 | 0.030 | 1.202 |  | >50 | 0.051 | ND | 0.045 |  | 0.013 | 0.015 | 0.120 |  |
|  |  | NIH45-46 | 0.219 | 0.864 | 0.983 | 0.089 | 2.444 |  | >50 | 0.224 | 0.066 | 0.138 |  | 0.045 | 0.027 | 0.170 |  |
|  |  | 3BNC60 | 0.240 | 1.208 | 0.318 | 0.367 | 4.736 |  | >50 | 0.129 | 0.038 | 0.092 |  | 0.057 | 0.081 | 0.130 |  |
|  |  | 3BNC117 | 0.104 | 1.277 | 0.214 | 0.174 | 4.713 |  | >50 | 0.121 | 0.062 | 0.069 |  | 0.102 | 0.054 | 0.084 |  |
|  |  | 12A12 | 0.448 | 3.180 | 0.406 | 0.150 | 20.500 |  | >50 | 0.205 | 0.107 | 0.360 |  | 0.037 | 0.067 | 0.842 |  |
|  |  | VRC-CH31 | 0.147 | 0.629 | 1.409 | 0.484 | 5.291 |  | >50 | 0.254 | 0.058 | 33.900 |  | 0.057 | 0.146 | 0.209 |  |
|  |  | VRC-PG04 | 0.746 | 3.627 | 1.568 | 0.259 | 4.417 |  | >50 | 0.410 | 0.254 | 29.215 |  | 0.100 | 0.238 | 0.313 |  |
|  |  | VRC-PG20 | 0.171 | 0.557 | 19.135 | 0.041 | 1.864 |  | >50 | 7.479 | 0.064 | 0.608 |  | 0.040 | 0.031 | 0.117 |  |
| V1V2 | | PG9 | >50 | 0.216 | 0.364 | 0.012 | 1.024 |  | 0.400 | 19.630 | 0.024 | 0.019 |  | 0.522 | 6.871 | 1.042 |  |
|  |  | PG16 | >50 | 0.061 | 0.787 | 0.005 | 4.260 |  | 8.742 | >50 | 0.002 | 4.302 |  | 0.509 | >50 | 0.329 |  |
|  |  | PGT145 | 2.551 | 0.093 | 2.632 | 0.004 | >50 |  | >50 | 0.193 | 0.001 | >50 |  | 0.079 | 13.590 | 0.816 |  |
|  |  | VRC26.25 | >50 | >50 | >50 | 0.002 | >50 |  | >50 | >50 | ND | >50 |  | 0.083 | >50 | 0.011 |  |
|  |  | PGDM1400 | 4.744 | 0.055 | 0.122 | 0.001 | 1.983 |  | 1.891 | 10.345 | ND | 0.042 |  | 0.419 | >50 | 0.019 |  |
| Glycan-V3 | | 10-1074 | >50 | >50 | >50 | >50 | >50 |  | 0.019 | 0.116 | ND | 0.020 |  | 1.714 | 0.019 | >50 |  |
|  |  | PGT121 | >50 | >50 | >50 | >50 | >50 |  | 0.026 | 0.100 | >50 | 0.009 |  | 0.292 | 0.030 | 0.042 |  |
|  |  | PGT128 | 45.020 | 0.019 | 0.029 | 0.015 | 15.485 |  | 0.010 | 0.011 | >50 | 0.008 |  | >50 | 0.015 | 0.021 |  |
|  |  | PGT135 | >50 | 1.552 | 1.720 | >50 | >50 |  | 3.581 | 0.440 | >50 | 0.079 |  | >50 | 0.055 | 0.118 |  |
| OD-glycan | | 2G12 | >50 | >50 | 0.175 | >50 | >50 |  | 2.583 | 0.729 | >50 | 0.198 |  | >50 | >50 | >50 |  |
| Fusion peptide | | PGT151 | >50 | >50 | >50 | >50 | >50 |  | 0.002 | 0.042 | >50 | >50 |  | 0.001 | >50 | >50 |  |
|  |  | VRC34.01 | >50 | >50 | 40.809 | >50 | >50 |  | >50 | >50 | ND | >50 |  | 0.359 | 6.580 | >50 |  |
| Subunit interface | | 35O22 | >50 | 0.333 | 0.017 | >50 | >50 |  | >50 | 0.001 | >50 | >50 |  | 0.001 | 0.348 | >50 |  |
| MPER | | 2F5 | 0.159 | 0.052 | 3.543 | 0.501 | 0.942 |  | 1.008 | 0.922 | 3.483 | 8.484 |  | >50 | >50 | >50 |  |
|  |  | 4E10 | 0.053 | 1.529 | 0.177 | 0.145 | 1.421 |  | 0.475 | 0.367 | 20.253 | 2.439 |  | 0.007 | 2.131 | 0.889 |  |
|  |  | 10E8 | 0.083 | 0.018 | 0.338 | 0.153 | 0.374 |  | 0.380 | 1.283 | 0.589 | 0.344 |  | 0.001 | 0.322 | 0.185 |  |
| Soluble CD4 | | CD4-Ig | 14.237 | 15.550 | 8.102 | 0.600 | >50 |  | 1.466 | >50 | 5.423 | 3.210 |  | 0.002 | >50 | 41.580 |  |
| **Negative control** | | | | | | | | | | | | | | | | | |
| Anti-Ebola | BB2G4 | | >50 | >50 | >50 | >50 | >50 |  | >50 | >50 | >50 | >50 |  | >50 | >50 | >50 |  |
| Anti-H5N1 | BB3C11 | | >50 | >50 | >50 | >50 | >50 |  | >50 | >50 | >50 | >50 |  | >50 | >50 | >50 |  |


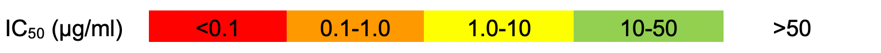


**Figure S1** Electrophoresis analysis of purified UFO trimers either under a reducing condition SDS-PAGE (**A**) or non-reducing native condition BN-PAGE (**B**). Various form of Envs and molecular weight markers are indicated. Reference-free two-dimensional (2D) class averages from negative-stain EM of three parental UFO trimers (**C**). As indicated by the 2D class averages, almost (>95%) of purified Env proteins appeared to be native-like trimers.

**
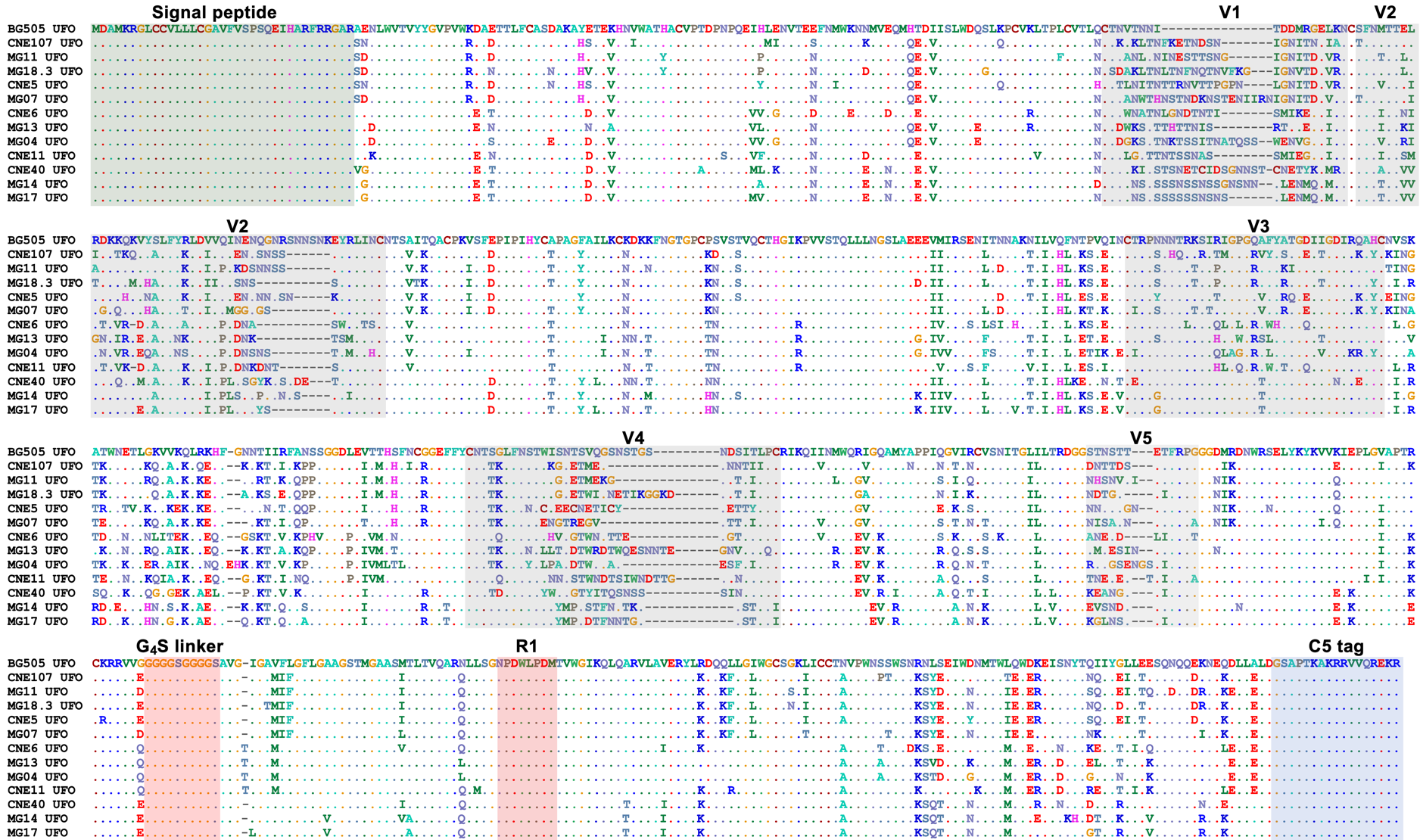
Figure S2** Sequence alignment of twelve selected envelope clones along with BG505 (reference) in UFO format. Signal peptide, hypervariable regions V1- V5, redesign of cleavage site (G_4_S linker) and N-terminus of HR1 (R1), and C5 tag are highlighted. Substitutions are denoted as upper-case letters. Dots represent the identical residues while dashes the deleted ones.
